# Optimized flip angle schemes for the split acquisition of fast spin‐echo signals (SPLICE) sequence and application to diffusion‐weighted imaging

**DOI:** 10.1002/mrm.29545

**Published:** 2022-11-24

**Authors:** Sofie Rahbek, Tim Schakel, Faisal Mahmood, Kristoffer H. Madsen, Marielle E.P. Philippens, Lars G. Hanson

**Affiliations:** ^1^ Department of Health Technology Technical University of Denmark Lyngby Denmark; ^2^ Department of Radiotherapy University Medical Center Utrecht Utrecht Netherlands; ^3^ Laboratory of Radiation Physics, Department of Oncology Odense University Hospital Odense Denmark; ^4^ Department of Clinical Research University of Southern Denmark Odense Denmark; ^5^ Danish Research Centre for Magnetic Resonance, Centre for Functional and Diagnostic Imaging and Research Copenhagen University Hospital Hvidovre Denmark; ^6^ Department of Applied Mathematics and Computer Science Technical University of Denmark Lyngby Denmark

**Keywords:** diffusion‐weighted magnetic resonance imaging, point spread function, SNR, SPLICE, sequence optimization, variable flip angle

## Abstract

**Purpose:**

The diffusion‐weighted SPLICE (split acquisition of fast spin‐echo signals) sequence employs split‐echo rapid acquisition with relaxation enhancement (RARE) readout to provide images almost free of geometric distortions. However, due to the varying T${}_{2}$‐weighting during k‐space traversal, SPLICE suffers from blurring. This work extends a method for controlling the spatial point spread function (PSF) while optimizing the signal‐to‐noise ratio (SNR) achieved by adjusting the flip angles in the refocusing pulse train of SPLICE.

**Methods:**

An algorithm based on extended phase graph (EPG) simulations optimizes the flip angles by maximizing SNR for a flexibly chosen predefined target PSF that describes the desired k‐space density weighting and spatial resolution. An optimized flip angle scheme and a corresponding post‐processing correction filter which together achieve the target PSF was tested by healthy subject brain imaging using a clinical 1.5 T scanner.

**Results:**

Brain images showed a clear and consistent improvement over those obtained with a standard constant flip angle scheme. SNR was increased and apparent diffusion coefficient estimates were more accurate. For a modified Hann k‐space weighting example, considerable benefits resulted from acquisition weighting by flip angle control.

**Conclusion:**

The presented flexible method for optimizing SPLICE flip angle schemes offers improved MR image quality of geometrically accurate diffusion‐weighted images that makes the sequence a strong candidate for radiotherapy planning or stereotactic surgery.

## 1 INTRODUCTION

Diffusion‐weighted (DW) MRI reflects the micro‐anatomy of tissues as it probes the local molecular mobility of water. This is particularly useful for cancer imaging as the high cellular density of tumour tissue restricts the water movement, which results in a high DW signal intensity of tumours compared to healthy tissue. Currently, the most common read‐out used in clinical DW‐MRI is single shot echo‐planar imaging (EPI). Fast k‐space traversal following a single excitation pulse ensures a low scan time minimizing the problem of motion artifacts. However, EPI is prone to geometrical distortions due to the high sensitivity to static field inhomogeneities. Therefore, the benefit of DW‐MRI contrast for tumour delineation in radiotherapy planning, for example, is compromised, especially when the region‐of‐interest is near an air cavity.^1^

Other DW‐MRI sequences combine diffusion‐encoding with a rapid acquisition with relaxation enhancement (RARE) readout.^2^ In this combination, it is important to address potential violations of the Carr Purcell Meiboom Gill (CPMG) conditions that the RARE acquisition is performed under. The CPMG conditions dictate the timing and phase relations of the refocusing pulses so they form coinciding stimulated and spin echoes with the same phase. However, the diffusion‐encoding in combination with subject motion essentially randomizes the initial phase of the echoes. This violates CPMG conditions, causing echoes to interfere destructively leading to severe artifacts.

**FIGURE 1:**
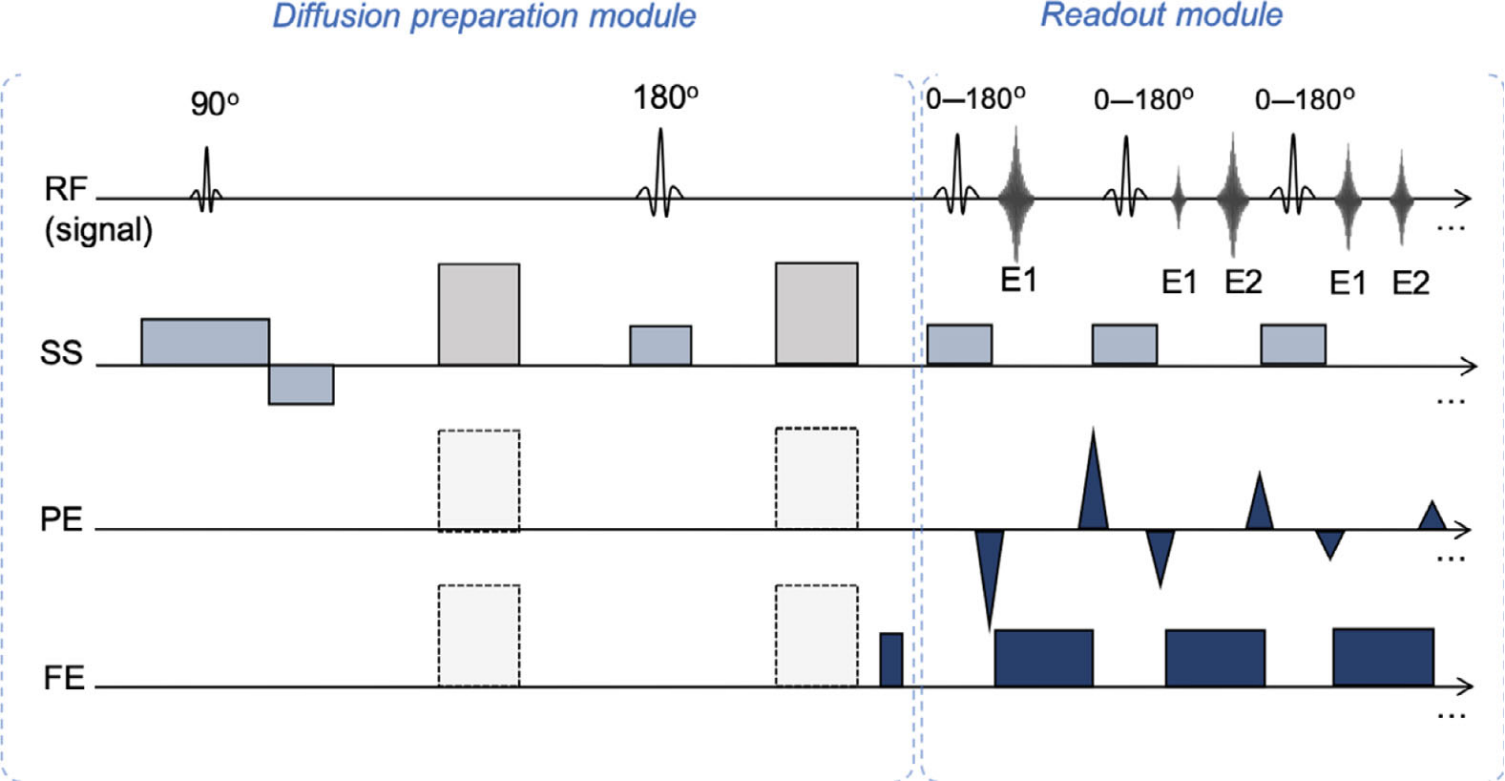
Schematic presentation of the diffusion‐weighted split acquisition of fast spin‐echo signals (SPLICE) sequence, where SS, PE and FE are the slice‐selection (SS), phase‐encoding (PE) and frequency‐encoding (FE) directions, respectively. The gray blocks are a diffusion‐encoding gradient pair (that can be applied in any direction), the light blue blocks are slice‐selective gradients, and the dark blue blocks are imaging gradients. The gradients in the FE direction are not balanced, as the pre‐phaser (gradient prior to the read‐out module) is much less than half the area of the following read‐out gradients. The two families of echoes appearing (E1 and E2) are illustrated on the top line together with the radiofrequency pulses. The flip angle is indicated for each pulse. The split‐echo mode implies that no phase relation for the refocusing pulses is required.^5^ For simplicity, crusher gradients are not shown.

Related to the work of Norris et al.^3^ and Alsop,^4^ Fritz Schick introduced the diffusion‐weighted split acquisition of fast spin‐echo signals (SPLICE) sequence,^5^ which overcomes the CPMG condition violation. In SPLICE, a prolonged and imbalanced read‐out gradient is used to split echoes with different phases into two families. Figure 1 illustrates the SPLICE sequence used in this study. It was originally published with a stimulated echo diffusion preparation.

Thus, two sets of echoes are collected, reconstructed, and combined as magnitude images. Other RARE‐based DW‐MRI sequences eliminate or suppress one of the echo families to overcome the CPMG violation, resulting in a more variable and potentially lower signal‐to‐noise ratio (SNR) than for SPLICE,^6^ depending on the phase introduced by slight motion. Alternatively, the SNR losses of these strategies can be reduced by introducing a quadratic phase relation for the refocusing pulses,^7^ but there is limited flexibility regarding refocusing flip angles, and if echo trains are long, severe blurring results. The geometrical robustness of the RARE‐based SPLICE makes it a compelling rival to echo‐planar imaging‐based DW‐MRI, especially when accurate anatomical delineations are critical.^8^ However, the longer signal sampling period causes pronounced blurring due to T${}_{2}$‐decay during k‐space traversal.

A strategy to reduce blurring and improve image quality is to modify the signal modulation by acquisition weighting^9^ using a variable refocusing flip angle in the echo train. The extended phase graph (EPG) algorithm^10^ calculates the magnetization response during a multipulse experiment and is useful for determining the resulting k‐space weighting for a RARE or SPLICE readout. Earlier work^11^, ^12^, ^13^, ^14^ utilized the prospective EPG formulation introduced by Hennig et al.^15^ to address the inverse problem of finding the flip angle scheme that generates a desired relaxation time contrast, and further used static pseudo steady states to control the signal decay. This strategy resulted in improvements, for example, low radiofrequency (RF) power deposition and reasonable point spread functions (PSFs) for a given contrast, but it did not optimize the SNR directly for a given PSF. Zhao et al.^16^ proposed a method for optimizing the flip angle scheme with a main focus of improving the SNR for a chosen spatial resolution in the phase‐encoding direction, expressed via the PSF (controlled blurring). We employ and extend this method for SPLICE diffusion‐weighted measurements that benefit from recording of both CPMG and non‐CPMG components, and where a main focus is SNR. Hence, there is limited need for controlling the relaxation time contrast. The employed method avoids the analytical solution of Hennig et al.,^15^ which may have an imaginary outcome and need iterative regulation of a set of flip angle control points. Instead, the method provides an optimized refocusing scheme and a corresponding filter for postprocessing to compensate for remaining differences between the final signal modulation and the desired k‐space weighting.^16^ The method thus offers a flexible tool for measurement design and provides an optimized solution with respect to SNR for a chosen PSF. The strategy also facilitates comparison between refocusing schemes since partial volume effects are inherently made similar. We extend the method to SPLICE, validate and demonstrate the value with simulations, phantom scans and brain scanning of healthy volunteers. A preliminary account was reported as a conference abstract.^17^

## 2 METHODS

### 2.1 Optimization algorithm

A normalized target distribution, T(k), is defined to describe the k‐space density weighting in the phase encoding‐direction corresponding to the desired spatial PSF through the Fourier transform. The sinc‐shaped PSF for normal uniform weighting (constant T(k)) of central k‐space, is not desirable due to pronounced spatial side‐bands, for example. T(k) is freely selectable and given as input to the optimization algorithm together with tissue relaxation parameters (T${}_{2}$, T${}_{1}$) and sequence specifications (echo spacing [ESP], echo train length [ETL]). The events of a SPLICE readout are simulated using EPG calculations, from which the final echo response, I(k), is given by the signal magnitude sum of the two echo families (E1 and E2) for a spatial point source. Hence, I(k) represents the k‐space weighting introduced by the flip angle scheme, and it depends on both acquisition and tissue parameters. As in an earlier publication concerning spin labeling sequences,^16^ a filter, F(k), is calculated to compensate for the differences between I(k) and T(k) to ensure the desired PSF in the resulting images (Equation 1). For normal linear image reconstruction and fixed PSF, the SNR only depends on the flip angle scheme via the filter's effect on noise (Equation 2).

(1) T(k)=I(k)·F(k).

(2) $$\text{SNR}\propto \frac{1}{\sqrt{\sum_{k}{\left(\frac{T(k)}{I(k)}\right)}^{2}}}.$$

The algorithm maximizes this SNR by updating the flip angle scheme using a nonlinear programming solver utilizing an interior‐point method (MATLAB 2018b, MathWorks, Inc.). The output is the optimized set of flip angles together with the final filter, preferably relatively flat as the echo response then already matches the desired signal weighting.^9^ Strong filters are undesirable due to decreased SNR and the user must therefore choose a reasonable PSF (voxel size and shape as exemplified below) considering the application at hand. The algorithm makes it possible to compare choices theoretically, and it provides an optimal refocusing scheme for each. The filter ensures that the target PSF is met for the chosen tissue parameters, which enables direct SNR comparisons. Software with examples is available at https://github.com/sofierahbek/flip‐angle‐optimization.

For the optimization, a standard EPG framework is used with relaxation values representing brain tissue at 1.5 T (T${}_{1}$ = 900 ms, T${}_{2}$ = 95 ms). Effects of finite‐duration RF pulses are neglected.^10^ We therefore investigated whether off‐resonance effects across the slice‐profile are negligible for a SPLICE sequence with a long echo train and a realistic RF pulse shape, using the open‐source JEMRIS MRI simulator.^18^ Additionally, JEMRIS was used to examine the build‐up of signal that is not diffusion‐encoded, a possible consequence of unwanted coherence pathways generated by the repeated refocusing pulses. Finally, the k‐space signal weightings obtained with JEMRIS simulations, EPG simulations and phantom scans were compared to verify that the simulations are realistic and thus useful for optimization.

### 2.2 The choice of the target function

**FIGURE 2:**
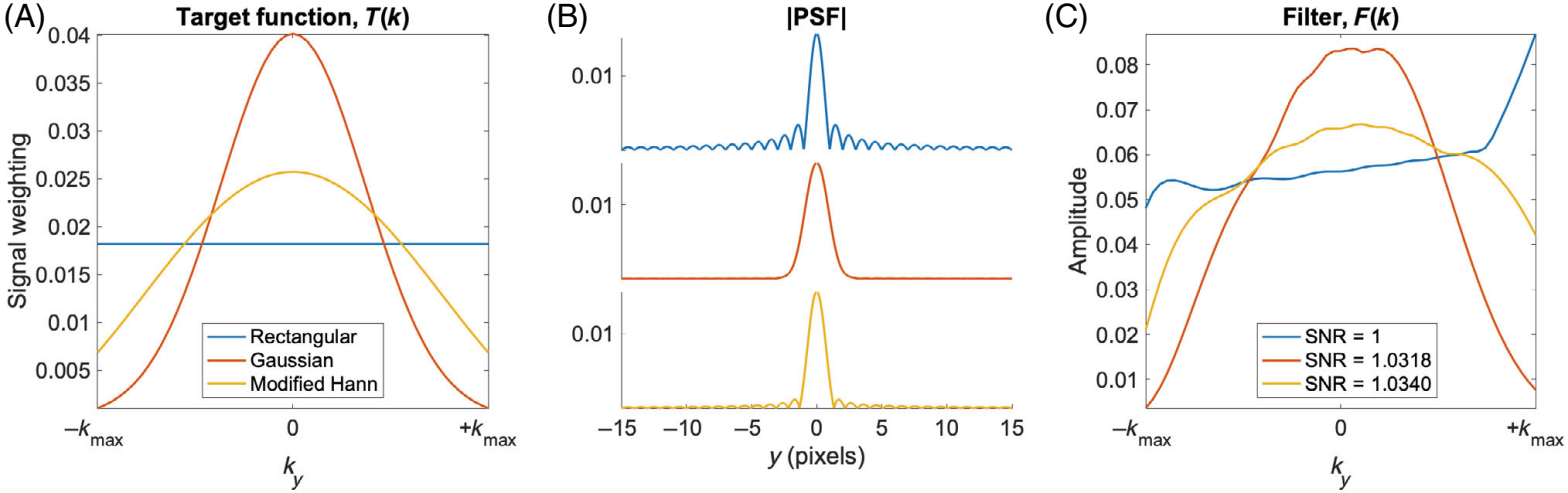
(A) Three different designs for the target function, all normalized so $\sum_{k}T(k)=1$. (B) The corresponding point spread function (PSF) calculated by Fourier transformation. (C) The filters needed to obtain the target functions after optimizing the flip angles for a linear k‐space sampling order. The specified signal‐to‐noise ratios are calculated by Equation (2) and normalized by the value for the rectangular weighting function.

Inspired by Pohmann et al.,^19^ an example target function (T(k)) was chosen as a modified Hann window defined as $w(k_{n})\propto 1+\cos(\frac{2\pi \cdot k_{n}\cdot \Delta y}{\alpha })$, with Δy being the nominal spatial resolution and the constant α set to 1.5. The resulting PSF has both a small full width at half maximum (FWHM) and suppressed sidebands. Figure 2 illustrates this by comparing PSFs corresponding to the modified Hann, rectangular, and Gaussian functions, respectively.

### 2.3 Data acquisition and reconstruction

Three human in‐vivo brain scanning sessions (scans 1–3) were conducted using a 1.5 T MRI system (Ingenia, Philips Healthcare) and a multislice single‐shot SPLICE sequence modified to apply user‐defined flip angles. Two healthy adult subjects volunteered for the scanning (one subject twice on different days) after informed consent. For all SPLICE scans, ESP = 4.9 ms, in‐plane nominal resolution 1.98×1.98 ${\text{mm}}^{2}$, slice thickness 5 mm, slice gap 0 mm, 29 slices, b‐values [0, 800] ${\text{s/mm}}^{2}$, diffusion time 40 ms, and three discarded startup echoes. Three orthogonal directions and four averages were obtained for diffusion‐weighted data. Measurements were conducted with optimized schemes of variable flip angles and a scheme of flip angles rapidly converging to 90 degrees [145, 90, 90,… 90] (default setting by the scanner vendor used as a rather arbitrary reference). For each case, a dataset was recorded with a fully sampled k‐space, with a parallel imaging (PI) sampling scheme, and with a PI and partial Fourier (PF) imaging sampling scheme, respectively. A linear k‐space sampling order, a PI acceleration factor of 2 and a PF factor of 0.6 was used. Table 1 shows timing parameters for each case. Raw k‐space data was extracted from the scanner to be able to apply the filters calculated in the optimization procedure. The data were then reconstructed using MATLAB 2018b with help from the Berkeley Advanced Reconstruction Toolbox^20^ and a Projection onto Convex Sets algorithm.^21^

**TABLE 1:** SPLICE sequence parameters

Sampling	ETL	TE (ms)	TR (ms)	Scan time (s)
Full	110	328	10,525	295
PI	55	193	7892	221
PI + PF	34	86	6762	189

Abbreviations: ETL, echo train length; PI, parallel imaging; PF, partial Fourier; TE, echo time; TR, pulse repetition time.

A multishot RARE sequence was included in the protocol to generate high‐resolution reference images used for tissue segmentation. A white‐matter (WM) mask was created using the Statistical Parametric Mapping toolbox (SPM12, version 7487)^22^ and used for quantitative evaluation.

### 2.4 Slice‐profile investigation

The slice‐profile, which depends on the refocusing pulse design (duration, shape, sidelobes, etc.) have imperfections that may accumulate throughout the RARE readout. Severe imperfections implying significant phase and flip angle variations across a slice could make the EPG calculations an inaccurate representation of the actual signal. Hence, The JEMRIS simulator was used to simulate the standard SPLICE sequence as implemented by the scanner vendor, though leaving out the diffusion‐weighting module. Relevant simulation settings are: T_2_ = 100 ms, T_1_ msub = 1000 ms, ESP  =  5.2 ms, ETL  =  70, flip angles: [156, 127, 120, 120,… 120]°. Phantom experiments for validating consistency between simulations and real data are described in Appendix S1.

Having two echo families, as is the case with the SPLICE sequence, complicates the slice profile investigation. Since absolute images from each echo family are combined in the reconstruction, a normal slice profile is not meaningful, except for the two echo families separately. When separated, the profiles provide little insight into the combined spatial variation of sensitivity, however. Hence, an effective slice profile is here defined to be the position‐dependent sum of the two absolute transversal magnetizations, that is, a measure of the sensitivity to magnetization in each position.

## 3 RESULTS

**FIGURE 3:**
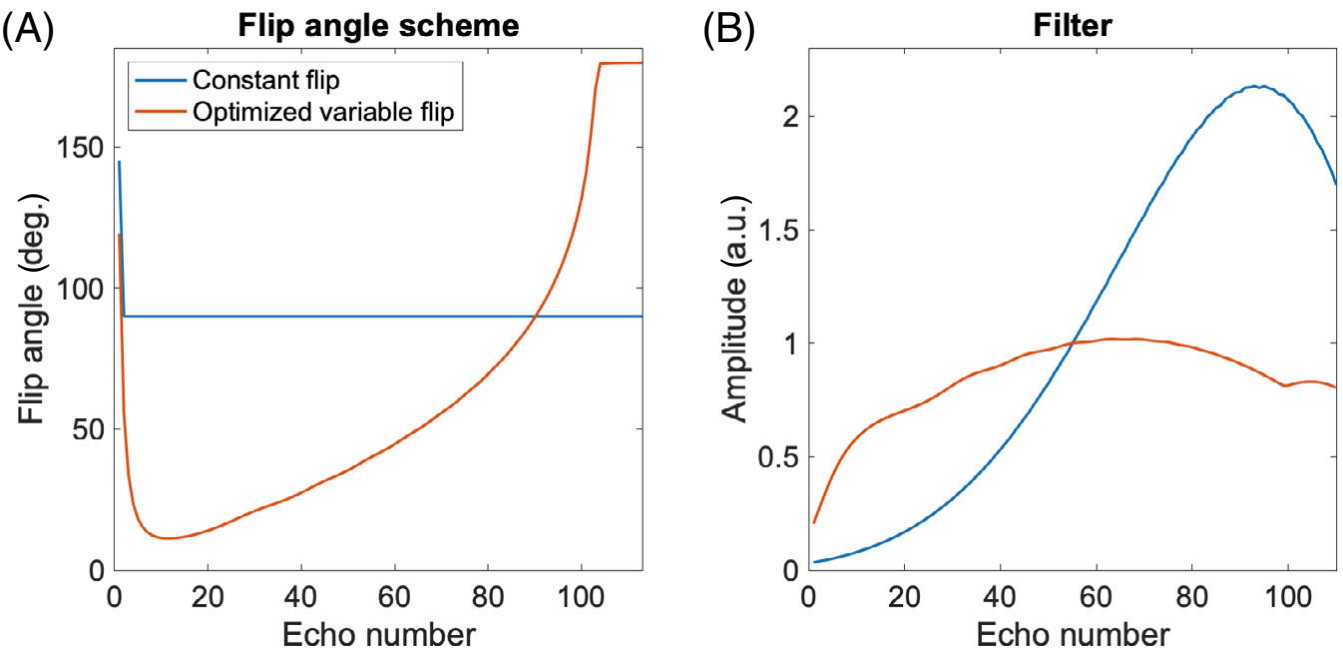
(a) Two flip angle schemes, the optimized (red) and a constant (blue). The optimized scheme is obtained using the settings: ETL = 110, ESP = 4.9 ms, T${}_{1}$ = 900 ms, T${}_{2}$ =  95 ms. (b) The corresponding compensation filters are normalized to have value 1 in the k‐space center. The relative flatness of the filter for the variable flip angle scheme indicates an SNR benefit of optimization.

The optimized flip angle scheme for the fully sampled data (linear k‐space sampling order, modified Hann window) is presented together with the compensation filter in Figure 3. The filter varies considerably, but is relatively flat compared to the filter needed for the reference flip angle scheme of repeated 90° pulses, shown for comparison. For the last eight echoes, the flip angle is 180°, indicating that a depletion of the longitudinal magnetization (so‐called z‐storage) is effectively reached. The flip angle schemes and filters for the undersampled cases can be found in Figure S1.

**FIGURE 4:**
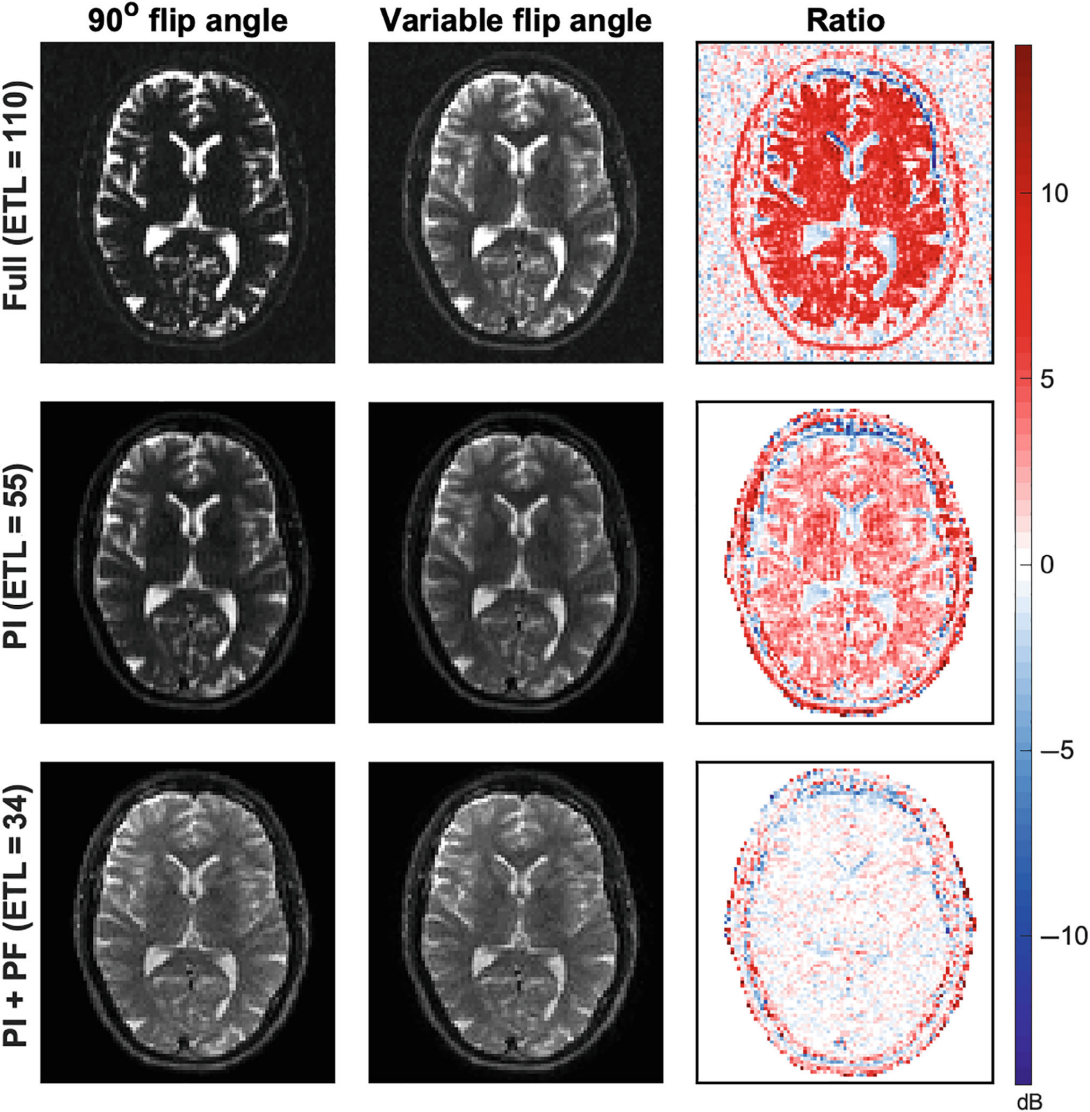
An example slice from scan 1 for the SPLICE *b* = 0 data scaled by the sum‐of‐squares background noise level. The constant flip angle data, the variable flip angle data, and the ratio in dB between those (which should be a measure of relative signal‐to‐noise ratio (SNR) given the noise scaling) are presented in the left, center, and right column, respectively. Red colors (positive values) represent a signal increase (SNR gain) for the variable flip angle data, and the opposite accounts for the blue colors (negative values). The fully sampled data is presented in the top row, and the undersampled data in the two bottom rows. Coil sensitivities were estimated only for areas within the subject, so the background is removed for undersampled data.

In Figure 4, the SPLICE *b* = 0  images (scan 1) are presented for a comparison between the two flip angle schemes. The magnitude images are scaled with the background noise in the (undersampled) sum‐of‐squares reconstructed images so that the visible intensity differences within a row reflect the SNR variation that is also directly shown in the third column. The introduction of optimized flip angles has changed the signal intensity and contrast of the images, especially for the fully sampled data. The *b* = 800 s/mm${}^{2}$ data for all scans are presented in Figure S6. The ratio images in the third column, showing the voxel‐to‐voxel relative signal change between the two datasets, make it clear that the SNR of brain matter has been improved using the optimized variable flip angles. The mean (± SD) SNR improvement in the WM mask across the three scans is a factor of 1.95 (± 0.066), 1.39 (± 0.029), and 1.03 (± 0.013) for the fully sampled, PI, and PI with PF data, respectively. More specifically, these values are calculated as the mean signal ratio across the WM region‐of‐interest in the noise‐scaled images (corresponding to a relative SNR improvement), and then the mean and SD across scans are given. Thus, the SNR gain increases for longer ETLs. Contrary to the brain matter, the SNR of the cerebrospinal fluid (CSF) is slightly reduced.

**FIGURE 5:**
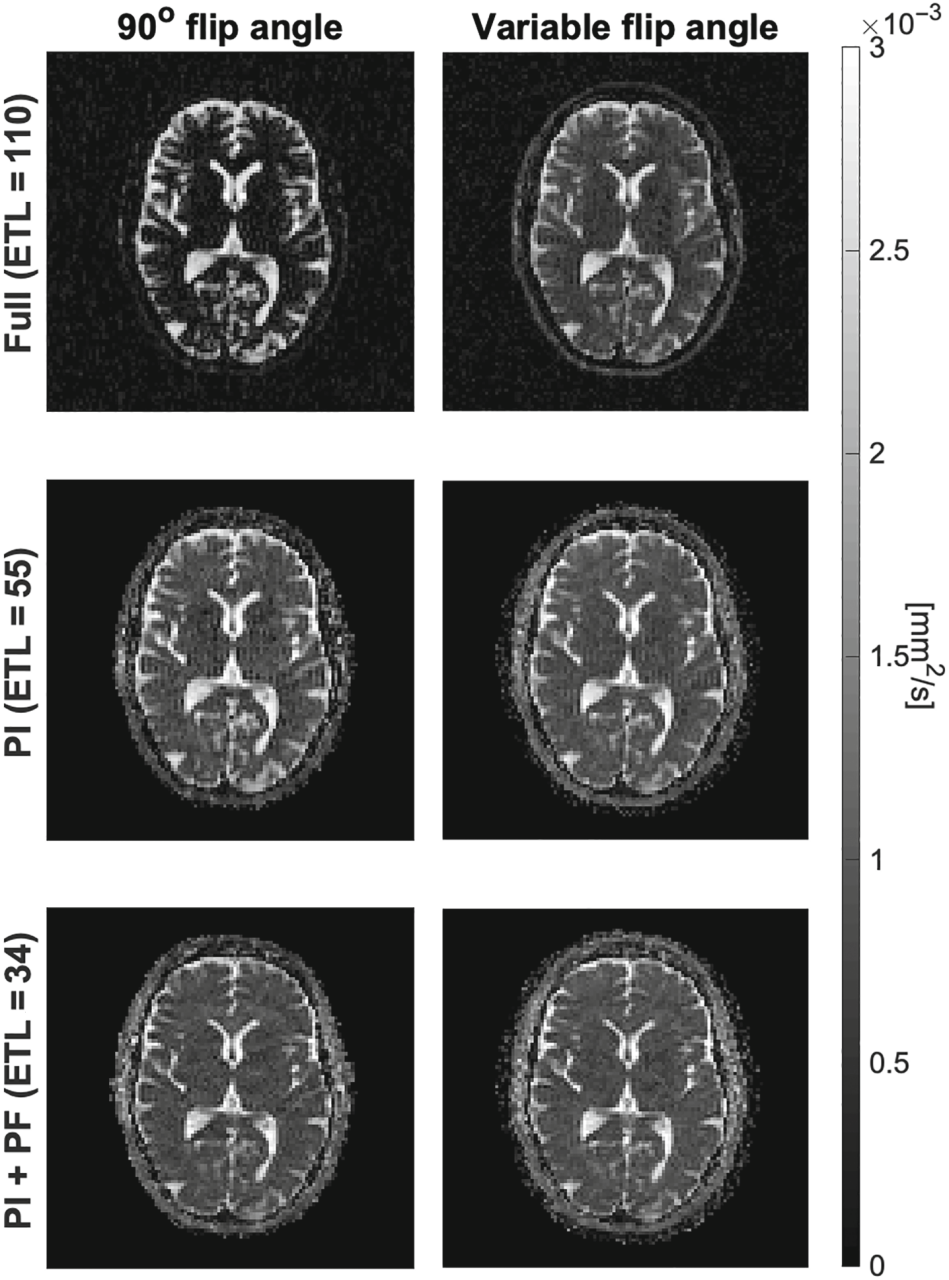
Apparent diffusion coefficient (ADC) maps for the SPLICE imaging data, same subject (scan 1) and slice as visualized in Figure 4. The left and right maps are for the constant and variable flip angle data, respectively. The mean ADC values in white‐matter for the fully sampled data (top row): $0.35\times 10^{-3}$ ${\text{mm}}^{2}$/s (left) and $0.73\times 10^{-3}$ ${\text{mm}}^{2}$/s (right), for the PI sampled data (middle row): $0.90\times 10^{-3}$ ${\text{mm}}^{2}$/s (left) and $0.95\times 10^{-3}$ ${\text{mm}}^{2}$/s (right), and for the PI and PF sampled data (bottom row): $0.89\times 10^{-3}$ ${\text{mm}}^{2}$/s (left), $0.95\times 10^{-3}$ ${\text{mm}}^{2}$/s (right)

Apparent diffusion coefficient (ADC) maps in Figure 5 show a generally higher ADC value in brain matter for the data recorded with the optimized variable flip angle scheme, especially considering the fully sampled data. Here, an average ADC value in the WM mask was estimated to $0.35\times 10^{-3}$ mm${}^{2}$/s and $0.73\times 10^{-3}$ mm${}^{2}$/s for the constant and variable flip angle data, respectively. The latter is comparable to reported ADC values based on high‐SNR data.^23^, ^24^

**FIGURE 6:**
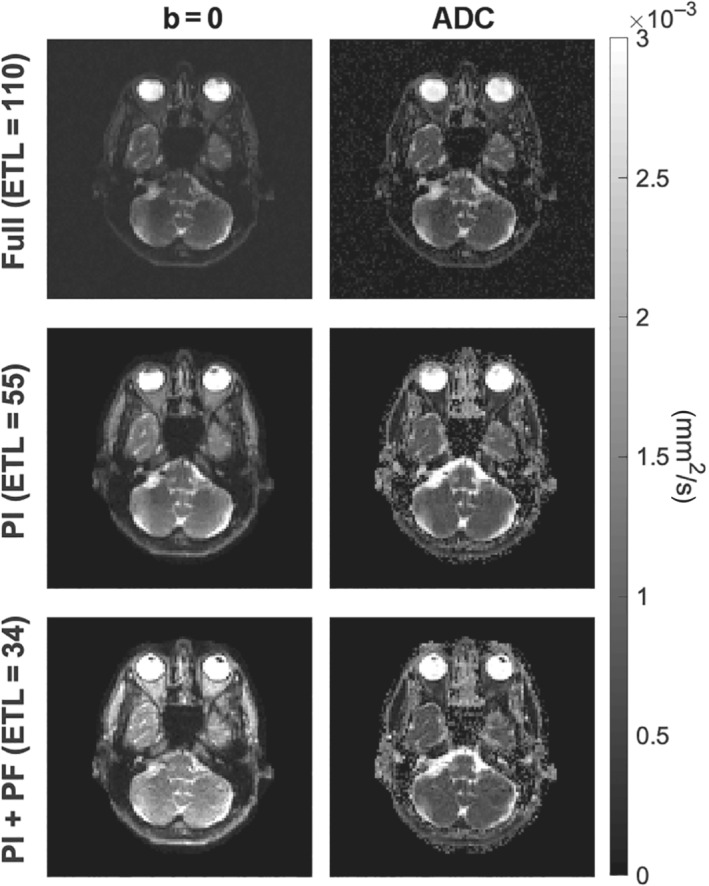
T${}_{2}$‐weighted b = 0 images and ADC maps of a low brain slice for one example dataset (scan 1, variable flip angle scheme). The fully sampled data is presented in the top row, and the undersampled data in the two bottom rows. The images are consistent across acquisitions and apparently free of motion artifacts.

In Figure 6, *b* = 0  and ADC maps for a slice at the base of the brain are shown for an example dataset obtained with optimized variable flip angles. In this part of the head, pulsatile, and respiratory movement is inevitable, but movement artifacts are not visible. This includes CSF signal voids that could potentially result from the temporary use of low flip angles, here down to around 10 degrees.^25^ Structure is seen within the brain tissue, but this is largely identical between the recordings with different refocusing schemes, and is therefore not motion artifacts, but consistent with expected tissue heterogeneity. Equivalent figures with data from the two other scan sessions reveal that results are consistent across scans, also in lower brain regions (Figures S5–S8).

**FIGURE 7:**
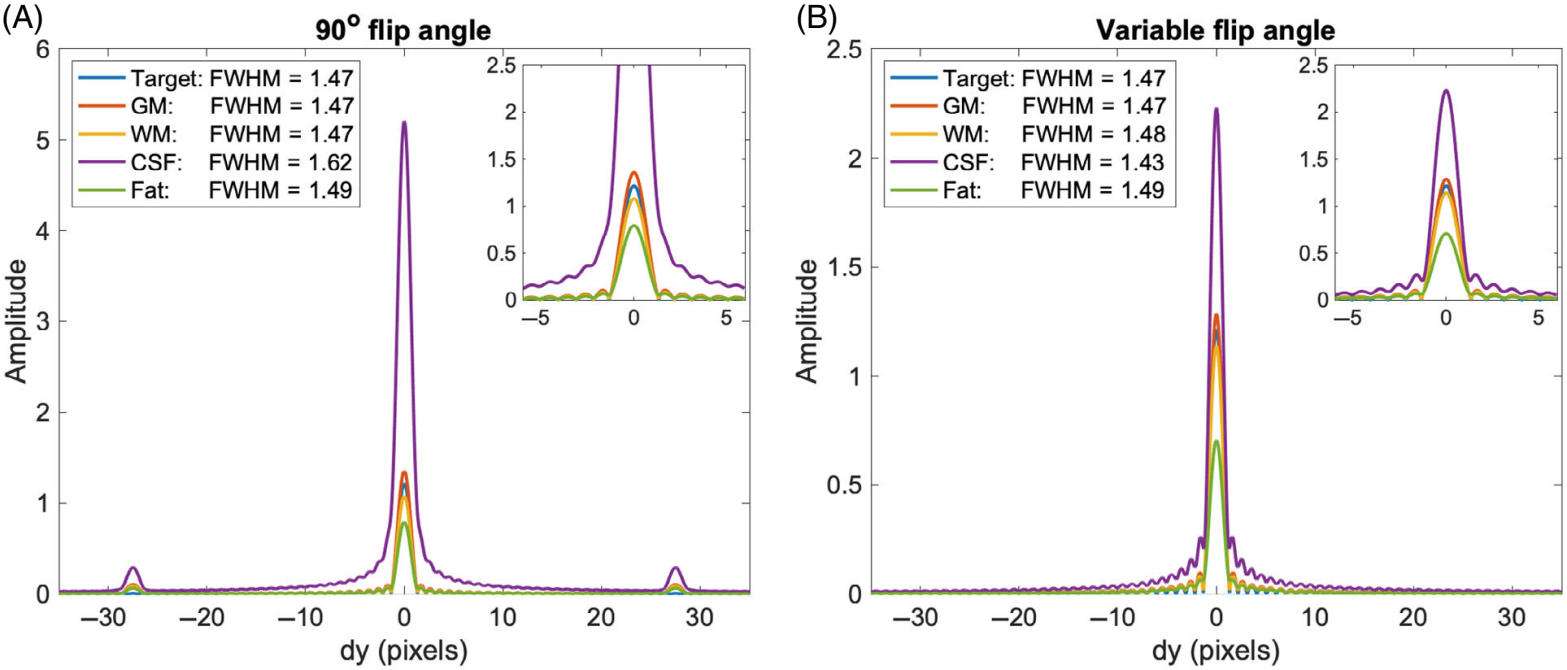
The point spread functions (PSFs) for different tissues after applying the correction filters of Figure 3: target brain tissue (T${}_{1}$ = 900 ms, T${}_{2}$ = 95 ms), GM (T${}_{1}$ = 1000 ms, T${}_{2}$ = 100 ms), white matter (T${}_{1}$ = 800 ms, T${}_{2}$ = 90 ms), cerebrospinal fluid (T${}_{1}$ = 2000 ms, T${}_{2}$ = 250 ms), and fat (T${}_{1}$ = 300 ms, T${}_{2}$ = 85 ms). The PSFs are presented for the 90° flip angle scheme (A) and the optimized variable flip angle scheme (B). The full width at half maximums of each PSF are specified in the legends. The similar PSFs for 10% reduced flip angles are provided in Figure S4.

The PSFs for other tissues than the target are not controlled via the optimization method and may be affected by the compensation filter. Figure 7 shows that the FWHM and shape of the PSF remained relatively unchanged when using the variable flip angle scheme, while a clear increase of the FWHM for the PSF of CSF is seen for the reference scheme of repeated 90° pulses, as well as two small “ghost” peaks. These results remained effectively unchanged after introducing a flip angle error of 10% and were thus robust to RF inhomogeneities (Figure S4).

**FIGURE 8:**
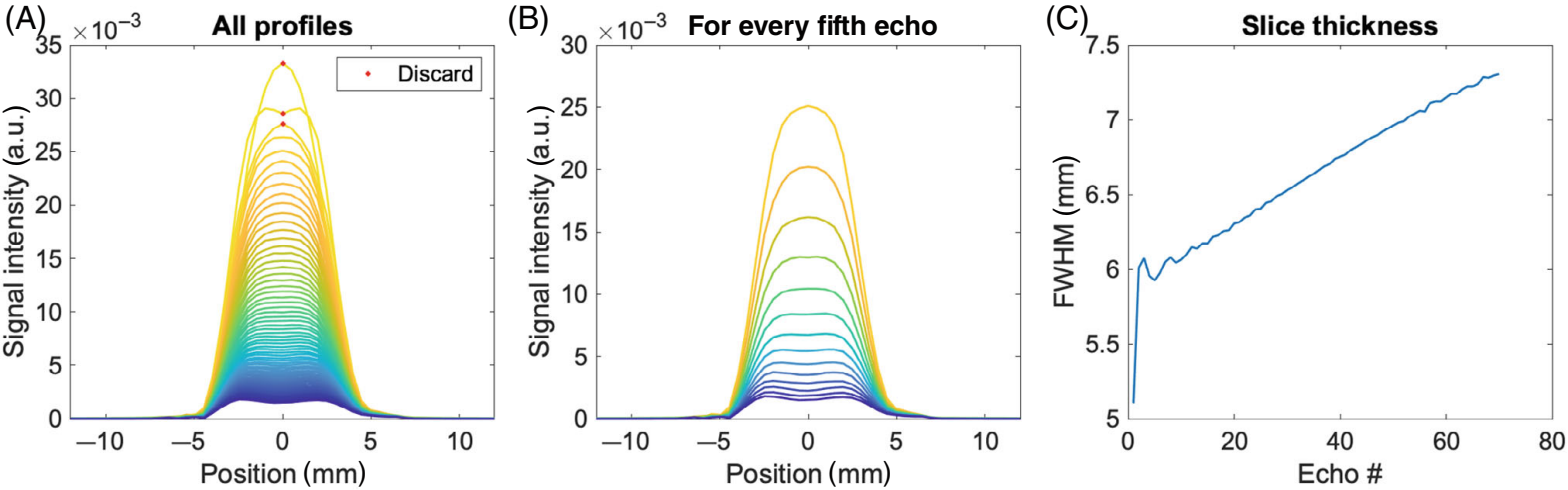
(A) All 70 slice‐profiles and (B) every fifth of these. The slice thickness calculated as full width at half maximum for all echoes (C). The first three echoes, denoted by red dots in (A), are recommended discarded due to the initial instability.

The results of the slice‐profile investigation using the JEMRIS simulator are presented in Figure 8. Initial transients occurred for the first three echoes, but otherwise the slice‐profile appeared relatively stable and without severe imperfections. The slice profiles for the two individual echoes recorded for each refocusing pulse differ (not shown), as can be expected since they represent different coherence pathways, and hence tend to vary oppositely as a function of position across the slice: In one position along the slice selection direction, the stimulated echoes may contribute most signal, whereas the spin echoes may give most signal in another position (due to the flip angle variation along that direction). Conveniently, the sum sensitivities for the two echo families, and therefore the effective slice profile, tends to be smooth. It is also seen to be well‐behaved over echoes in the sense that the slice width remains relatively constant during the echo train, except for the initial echoes (indicated by red dots).

This investigation was also performed for a flip angle scheme with more variation (not shown). Results were similar to the presented example to the extent that conclusions were the same. The JEMRIS simulations thus indicate that EPG calculations can be used as a good approximation of the signal in a flip angle optimization when the first three echoes are discarded.

The build‐up of unwanted steady‐state signal was very low (<2.1%) during the entire echo train compared to the desired attenuated diffusion‐weighted signal (Figure S2). Additionally, the figure shows that only small oscillations appeared in each echo family although a relatively large flip angle was used in the simulation (120°). This warrants that E1 and E2 are not considered individually, but only summed in the optimization algorithm. Finally, the expected consistency between simulations and MRI phantom scans was found (Figure S3).

## 4 DISCUSSION

The suggested optimized variable flip angle scheme resembles designs presented in earlier work where signal stabilization during long echo trains was explicitly established.^13^, ^25^, ^26^ This indicates that our solutions, which are optimal with respect to SNR for the given PSF, may also be near‐optimal considering other image traits. The optimization for SNR offers flexibility in terms of choosing the target PSF, and though a filter may be needed to realize the chosen voxel shape, consideration of flip angle control points is not needed. Using the methods in References 12, 25, for example, three angles must be chosen.

The target function suggested by Pohmann et al., resulted in a comparatively flat compensation filter which indicates a high benefit of the acquisition weighting obtained by varying the refocusing flip angle. As the filter is only fully effective for tissue with the parameters used in the optimization algorithm, blurring or artifacts may occur for other tissues. Optimized schemes temporarily employing relatively low refocusing angles increase the robustness towards tissue differences and transmit field inhomogeneities.^26^, ^27^ Figure S4 demonstrates the robustness by presenting the PSF for different tissues and a 10% flip angle error. The PSF is relatively unchanged for all tissues using the optimized flip angle scheme, whereas the suboptimal 90° flip angle scheme results in a PSF for CSF with small “ghost” peaks and a large FWHM compared to the other tissues.

The validation test presented in Appendix S1 justified the use of simulations in the optimization algorithm. Even though JEMRIS simulations showed a better resemblance to actual MRI measurements, EPG simulations are computationally efficient, easy to implement and still provide reasonable estimates of the magnetization response. This is consistent with Weigel et al.^28^ who compared EPG simulations with clinical measurements and found less than 2% difference. Full simulations (e.g. JEMRIS) or extended versions of EPG, which takes into account the slice profile,^29^ are options, but our results do not show a need for more accuracy.

The SNR improvement seen in Figure 4 confirms that an optimized design of the flip angle scheme is effective in practice, and further that the gain is increased for longer echo trains. Importantly, the use of optimized flip angle schemes involves no significant trade‐offs. The lower gain for sequences with a relatively short ETL is expected, as the echo time is shorter and the undesired signal modulation caused by the T${}_{2}$‐weighted signal decay already is reduced by lowering the number of echoes needed for k‐space traversal. Using an optimized flip angle design leaves room for increasing the ETL to, for example, obtain a higher spatial resolution or avoid undersampling strategies without a severe SNR trade‐off.

While earlier work optimized the flip angle scheme primarily for standard RARE sequences, we have implemented the optimized scheme for the diffusion‐weighted SPLICE sequence and demonstrated its significance for ADC estimation (Figure 5). Except for CSF that has high SNR for either examined flip angle scheme at both b‐values (T${}_{2}$ shine‐through), the SNR improvement resulting from optimization, increased the accuracy of the ADC estimate over the reference sequence. The underestimation of ADC when constant refocusing angles are used, is due to the severe signal bias for low SNR magnitude data acquired at high b‐values (noncentral chi signal distribution). This strongly argues for adopting optimized flip angle designs when using RARE‐based DWI sequences. A related positive effect of the variable flip angle design is a reduction of T${}_{2}$ shine‐through by the inherent shortening of the contrast‐equivalent TE.^26^ This effect was evident for the fully sampled b = 800 s/mm${}^{2}$ data in Supporting Information as the image contrast of the suboptimal 90° flip angle sequence indicates a strong T${}_{2}$ contrast compared to the optimized sequence. A major motivation for focusing on SPLICE is its potential for DWI when geometric distortion is particularly problematic such as for radiotherapy planning where PSF sidebands in addition compromises quantitative analysis. To that end, the results presented in Figures 4, 5, 6 are promising. Despite the SPLICE focus triggered by clinical needs for improvement, similar strategies may also apply to other multi‐echo sequences as previously demonstrated for arterial spin labelling sequences.^16^ However, the focus on optimizing SNR without controlling relaxation time contrast is not ideal for most RARE applications.

The method was demonstrated with consistent results from three scan sessions (Figures S6–S8), including lower brain regions where motion is unavoidable due to cardiac pulsation and breathing (Figure S8). CSF signal voids or other motion‐related signal voids were not observed despite earlier reports of such when small refocusing flip angles are employed,^25^ as is temporarily the case for the optimized schemes. Relative robustness may be expected since motion during single‐shot sequences is limited and since SPLICE is designed for relative motion insensitivity (fewer coherence pathways interfere compared to RARE). The optimization that is independent from the diffusion‐weighting was performed under an assumption of linear reconstruction, but nonlinear methods such as compressed sensing are similarly expected to benefit from sampling schemes guided by the desired spatial resolution. The method is straight‐forward to implement (software is provided) and directly applicable on scanners where user‐defined flip angle schemes and filtering can be introduced. The specific choices of PSFs, echo train lengths, and k‐space sampling orders are examples only, and with the provided software, other choices can readily be explored, for example, guided by findings by Zhao et al.^16^ That study demonstrated somewhat similar optimized flip angle schemes despite important sequence differences that affect slice profiles, for example. An example of centric k‐space acquisition is provided in Figure S5D showing strong gains over nonoptimized sequences since the filter needed to avoid blurring is strongly noise‐amplifying. For optimized refocusing schemes, the sampling order of k‐space lines is of limited importance for SNR, despite the shortening of echo times for centric sampling. This results from the initially excited magnetization being partially stored longitudinally until measured, and hence subject to slow decay. For example, a limited SNR gain of 20% in the WM mask is observed for full centric sampling over linear sampling (consistent with the relative flatness of the corresponding filters in Supporting Information Figure S1A,D). Even limited SNR gains are worth considering. However, attention needs to be paid to the variation of the slice profile in the early echoes (Figure 8), and hence an increased need for discarding these for centric recording. Furthermore, even though the slice profile is relatively constant throughout the SPLICE echo train as shown in Figure 8, it does vary somewhat. Acquiring the k‐space center halfway through the echo train therefore makes the slice profile most similar for high and low spatial frequencies, thereby improving the spatial specificity.

## 5 CONCLUSION

Optimizing the refocusing flip angle scheme of the single‐shot SPLICE sequence improves image quality, in particular with respect to SNR and spatial specificity. This directly benefits DWI, but also reduces T${}_{2}$ shine‐through, ADC bias resulting from low SNR, and partial volume effects. Together with the improved geometric accuracy of SPLICE relative to echo‐planar imaging, this makes the diffusion‐weighted SPLICE attractive for radiotherapy planning, for example. The algorithms are readily available, and the sequence and reconstruction changes needed are only flip angle adaptations and filtering.

## Supporting information




**Appendix S1**. Supporting information
**Figure S1:** Flip angle schemes and filters used for the recorded data. The left column (a‐c) contains schemes and filters for a linear k‐space sampling order, while the right column (d‐f) contains schemes and filters for a center‐out k‐space sampling order. (a) and (d) are for the fully sampled case (ETL = 110), (b) and (e) are for the PI sampled case (ETL = 55), and (c) and (f) are for the PI plus PF sampled case (ETL = 34).
**Figure S2:** Comparisons of the DW SPLICE signal with the no‐excitation signal. (a) Slice‐profiles. (b) Echo signals. (c) The ratio between the echo signals, that is, the red and green curve in (b). A *b*‐value of 500 s/mm^2^ and ADC value of 800 × 10^−6^ mm^2^/s were used for the DW factor.
**Figure S3:** Comparison of the EPG simulation, the JEMRIS simulation, and the MRI phantom scan for a constant flip angle scheme (a) and a variable flip angle scheme (b). The curves represents the ratio between the raw k‐space signals from the two echo families, E1 and E2. For the EPG and JEMRIS simulations, the k = 0 signal is used, for the MRI phantom data, the square root of the signal power over the frequency encoding direction is used.
**Figure S4**: The PSFs for different tissues after applying the correction filters of Figure 3 in the main manuscript: target brain tissue (T_1_ = 900 ms, T_2_ = 95 ms), GM (T_1_ = 1000 ms, T_2_ = 100 ms), WM (T_1_ = 800 ms, T_2_ = 90 ms), CSF (T_1_ = ms, T_2_ = 250 ms), and fat (T_1_ = 300 ms, T_2_ = 85 ms). The PSFs are presented for the 90° flip angle scheme (a) and the optimized variable flip angle scheme (b), and again with an introduced RF error, corresponding to 10 % reduced flip angles in (c) and (d). The FWHMs of each PSF are specified in the legends.
**Figure S5:** An example slice for the SPLICE *b* = 0 s/mm^2^ data scaled with the background noise level for all three scans/subjects. (a): scan 1, linear k‐space sampling order (also presented in Figure 4 of the main manuscript). (b): scan 2, linear k‐space sampling order. (c): scan 3, linear k‐space sampling order. (d): scan 2, centric k‐space sampling order. All images are presented with a common, arbitrary intensity scale for each row of images. For each subfigure, the fully sampled data is presented in the top row, and the undersampled data in the two bottom rows. Coil sensitivities were estimated only for areas within the subject, so the background is removed for undersampled data.
**Figure S6:** A single slice for the SPLICE *b* = 800 s/mm^2^ data for all three scans/subjects (obtained with linear k‐space sampling order). (a): scan 1. (b): scan 2. (c): scan 3. Images are presented with a common, arbitrary intensity scale for each row of images. The fully sampled data is presented in the top row, and the undersampled data in the two bottom rows. Coil sensitivities were estimated only for areas within the subject, so the background is removed for undersampled data.
**Figure S7:** ADC map for a single slice for all three scans/subjects. (a): scan 1. (b): scan 2. (c): scan 3. The fully sampled data is presented in the top row, and the undersampled data in the two bottom rows.
**Figure S8:** (a) *b* = 0 s/mm^2^ images and (b) ADC maps of the lower brain for one example dataset (scan 1, variable flip angle scheme). The fully sampled data is presented in the top row, and the undersampled data in the two bottom rows. Slice 6 (right) is also shown in Figure 6 of the main manuscript.
