# Supplementary material for: Optimized flip angle schemes for the split acquisition of fast spin‐echo signals (SPLICE) sequence and application to diffusion‐weighted imaging
Source: Magn Reson Med. 2022 Nov 24;89(4):1469–80. doi: 10.1002/mrm.29545 (PMC10099388; doi:10.1002/mrm.29545)
Supplement: Supplementary file 1 — Appendix S1. Supporting information Figure S1: Flip angle schemes and filters used for the recorded data. The left column (a‐c) contains schemes and filters for a linear k‐space sampling order, while the right column (d‐f) contains schemes and filters for a center‐out k‐space sampling order. (a) and (d) are for the fully sampled case (ETL = 110), (b) and (e) are for the PI sampled case (ETL = 55), and (c) and (f) are for the PI plus PF sampled case (ETL = 34). Figure S2: Comparisons of the DW SPLICE signal with the no‐excitation signal. (a) Slice‐profiles. (b) Echo signals. (c) The ratio between the echo signals, that is, the red and green curve in (b). A b‐value of 500 s/mm2 and ADC value of 800 × 10−6 mm2/s were used for the DW factor. Figure S3: Comparison of the EPG simulation, the JEMRIS simulation, and the MRI phantom scan for a constant flip angle scheme (a) and a variable flip angle scheme (b). The curves represents the ratio between the raw k‐space signals from the two echo families, E1 and E2. For the EPG and JEMRIS simulations, the k = 0 signal is used, for the MRI phantom data, the square root of the signal power over the frequency encoding direction is used. Figure S4: The PSFs for different tissues after applying the correction filters of Figure 3 in the main manuscript: target brain tissue (T1 = 900 ms, T2 = 95 ms), GM (T1 = 1000 ms, T2 = 100 ms), WM (T1 = 800 ms, T2 = 90 ms), CSF (T1 = ms, T2 = 250 ms), and fat (T1 = 300 ms, T2 = 85 ms). The PSFs are presented for the 90° flip angle scheme (a) and the optimized variable flip angle scheme (b), and again with an introduced RF error, corresponding to 10 % reduced flip angles in (c) and (d). The FWHMs of each PSF are specified in the legends. Figure S5: An example slice for the SPLICE b = 0 s/mm2 data scaled with the background noise level for all three scans/subjects. (a): scan 1, linear k‐space sampling order (also presented in Figure 4 of the main manuscript). (b): scan 2, linear k‐space sampling orde [file MRM-89-1469-s001.pdf]

Supporting Information

Flip angle schemes and filters

Figure S1 presents the flip angle schemes and corresponding correction filters used for the recorded data. The filters are calculated using the modified Hann function of Figure 2 in the main manuscript as target function.

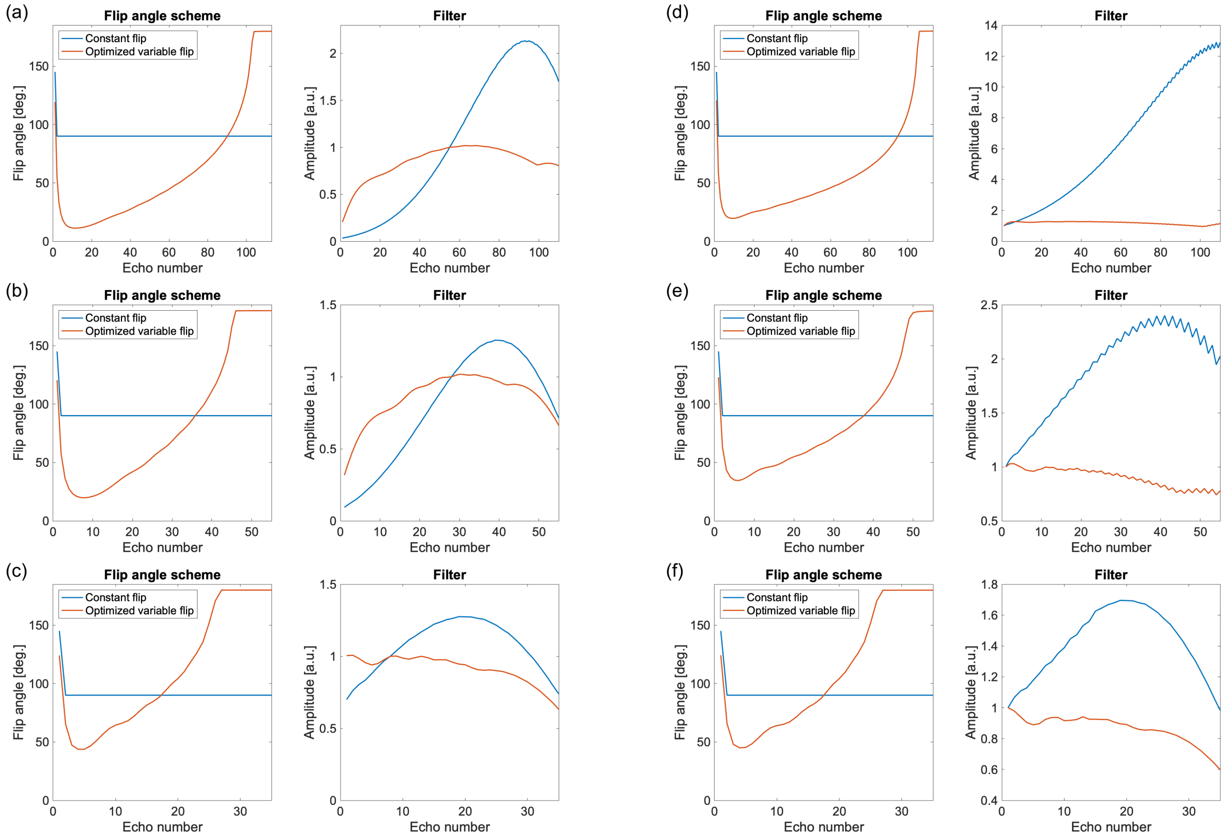

Figure S1: Flip angle schemes and filters used for the recorded data. The left column (a-c) contains schemes and filters for a linear k-space sampling order, while the right column (d-f) contains schemes and filters for a center-out k-space sampling order. (a) and (d) are for the fully sampled case (ETL=110), (b) and (e) are for the PI sampled case (ETL=55), and (c) and (f) are for the PI plus PF sampled case (ETL=34).

Steady state signal investigation

A build-up of non-diffusion-weighted steady state signal may occur as a consequence of unwanted coherence signal pathways. This problem was investigated by comparing the signal from a JEMRIS simulation *with* a 90° excitation pulse with one *without* excitation ("no excitation"). Diffusion-weighting of the excited magnetization was simulated by multiplying the JEMRIS output with a weighting factor ($e^{-b \cdot \mathrm{ADC}}$). Figure S2 shows a thorough comparison, presenting both the slice-profiles for the two cases as well as signal integrated over the slice profile (echo signal). Throughout the echo train, the steady state signal is low relative to the diffusion-weighted SPLICE signal, corresponding to approximately 2.1 % after 110 echoes.

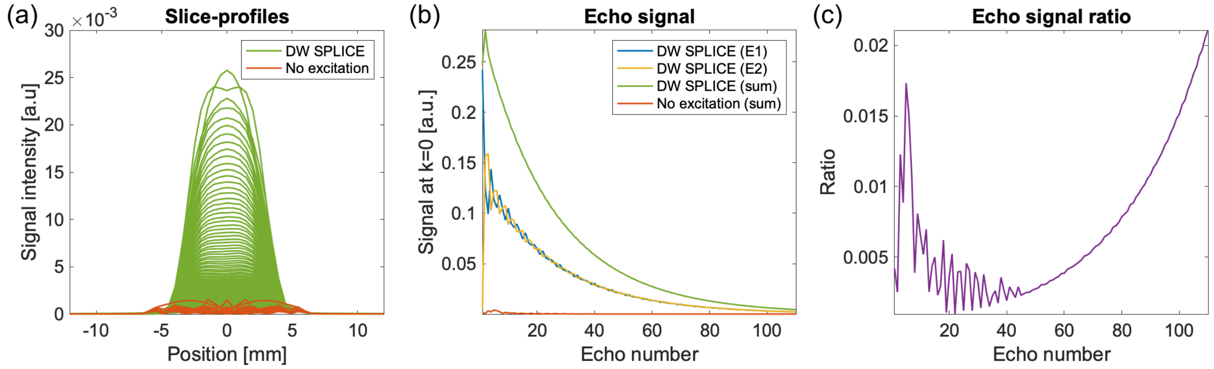

Figure S2: Comparisons of the DW SPLICE signal with the no-excitation signal. (a): Slice-profiles. (b): Echo signals. (c): The ratio between the echo signals, i.e. the red and green curve in (b). A b-value of 500 s/mm^2 and ADC value of 800×10^{-6} mm^2/s were used for the DW factor.

Figure S2(b) also shows the echo amplitude for the two echo families (E1, E2) separately. Only very small oscillations are present even though a relatively large flip angle (120°) is used throughout the echo train. Theoretically, a repeated large flip angle will shift a large portion of the signal between the two echo families for each echo resulting in oscillations in the individual echo families. However, the imperfections of the slice-profile reduce this behaviour and the oscillations are dampened. A JEMRIS simulation has confirmed this effect why oscillations within each echo family have been ignored in the flip angle optimization.

Validation of simulations

To validate that simulations are in agreement with actual MRI scans, the k-space weighting along the phase-encoding direction was compared for EPG simulations, JEMRIS simulations and MRI of a spherical phantom with a homogeneous center (PIQT phantom (Philips Healthcare, Best, The Netherlands), T_1=350 ms, T_2=320 ms) for two different flip angle schemes. For the SPLICE scan of the phantom, ESP=5.2 ms, in-plane nominal resolution 3.95x3.95 mm^2, slice thickness 5 mm, slice gap 5 mm, 20 slices, ETL=63, TE=226 ms, TR=4030 ms and scan time=64.5 s. Only b=0 s/mm^2 data was used, and from a single central slice. To eliminate the effect of the object, the ratio between the two echo families (E1/E2) was considered. The resulting oscillating pattern, stemming from a signal shift between echo families, was relatively consistent between simulations and phantom scans for both of the tested flip angle schemes (Figure S3). This consistency supports that simulations can be trusted in the optimization.

Robustness

Figure S4(c,d) illustrate the sensitivity of the PSF to 10% deviation between nominal and actual flip angles. This shows high robustness to RF inhomogeneity as the PSFs are almost unchanged relative to corresponding PSFs calculated without RF miscalibration ((a,b) repeated from main manuscript Figure 7 to ease comparison).

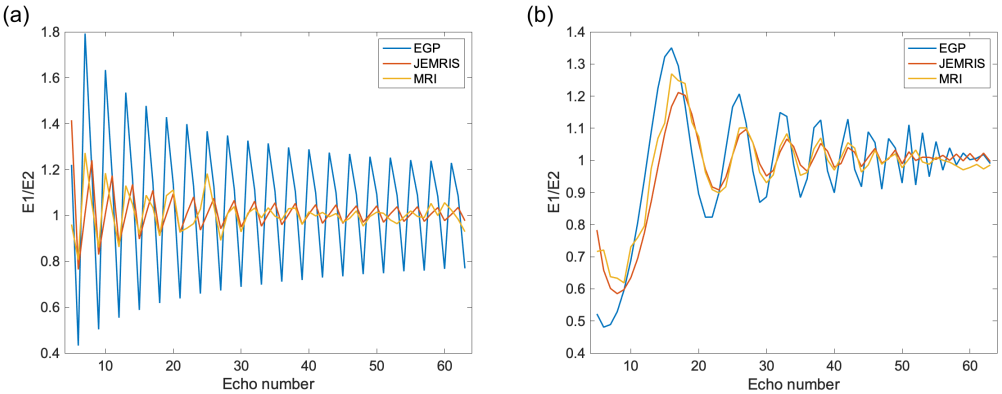

Figure S3: Comparison of the EPG simulation, the JEMRIS simulation, and the MRI phantom scan for a constant flip angle scheme (a) and a variable flip angle scheme (b). The curves represents the ratio between the raw k-space signals from the two echo families, E1 and E2. For the EPG and JEMRIS simulations, the k=0 signal is used, for the MRI phantom data, the square root of the signal power over the frequency encoding direction is used.

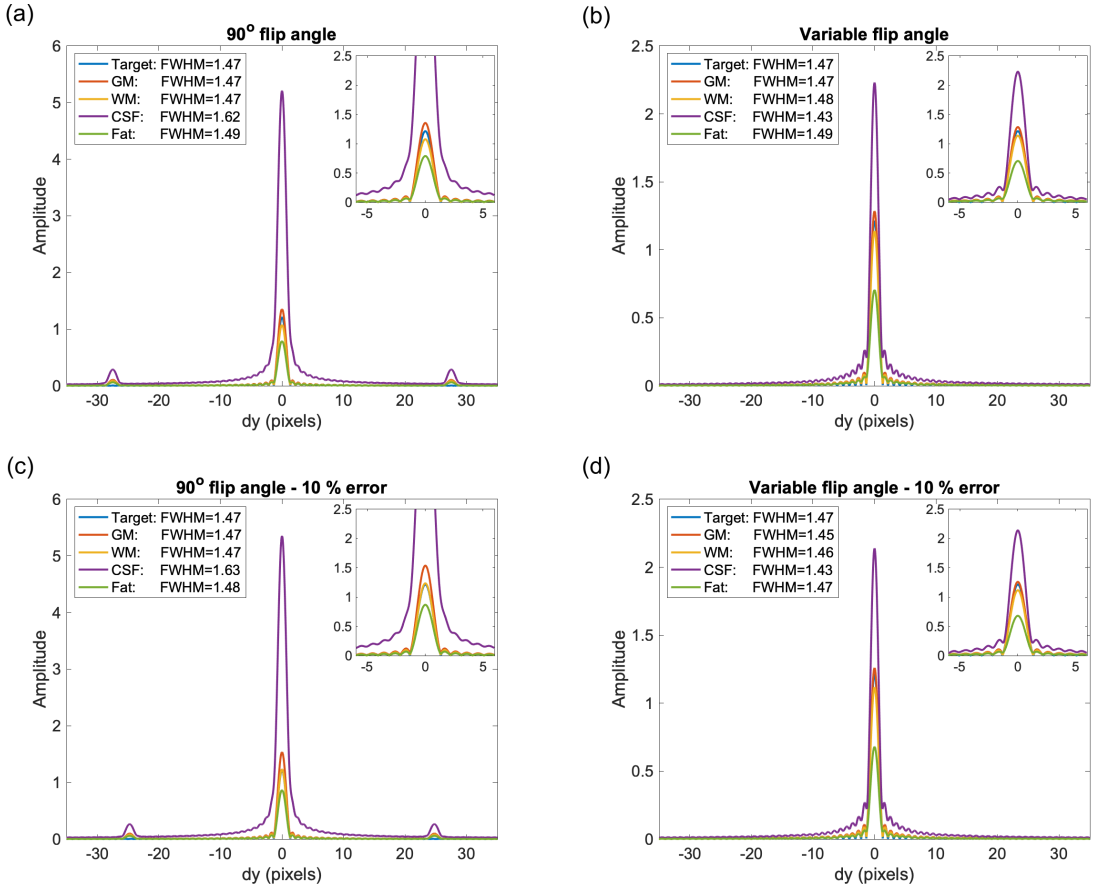

Figure S4: The PSFs for different tissues after applying the correction filters of Figure 3 in the main manuscript: target brain tissue (T_1=900 ms, T_2=95 ms), GM (T_1=1000 ms, T_2=100 ms), WM (T_1=800 ms, T_2=90 ms), CSF (T_1=2000 ms, T_2=250 ms), and fat (T_1=300 ms, T_2=85 ms). The PSFs are presented for the 90° flip angle scheme (a) and the optimized variable flip angle scheme (b), and again with an introduced RF error, corresponding to 10 % reduced flip angles in (c) and (d). The FWHMs of each PSF are specified in the legends.

Figure S5 shows the resulting b=0 s/mm^2 images for all three scans (recorded with a linear k-space sampling order), as presented in Figure 4 of the main manuscript. Additionally, example data recorded with a centric k-space sampling order is presented in subfigure S5(d). The image quality is notably low for the fully sampled data acquired with a non-optimized flip angle scheme during a long echo train. This is expected since the resulting strong filter tailored to avoid blurring of brain tissue (not CSF) causes severe noise amplification. Therefore also strong edge enhancement is seen for CSF. Such image degradation is not visible for the shorter echo-trains and for the optimized refocusing schemes that are designed to minimize the need for filtering.

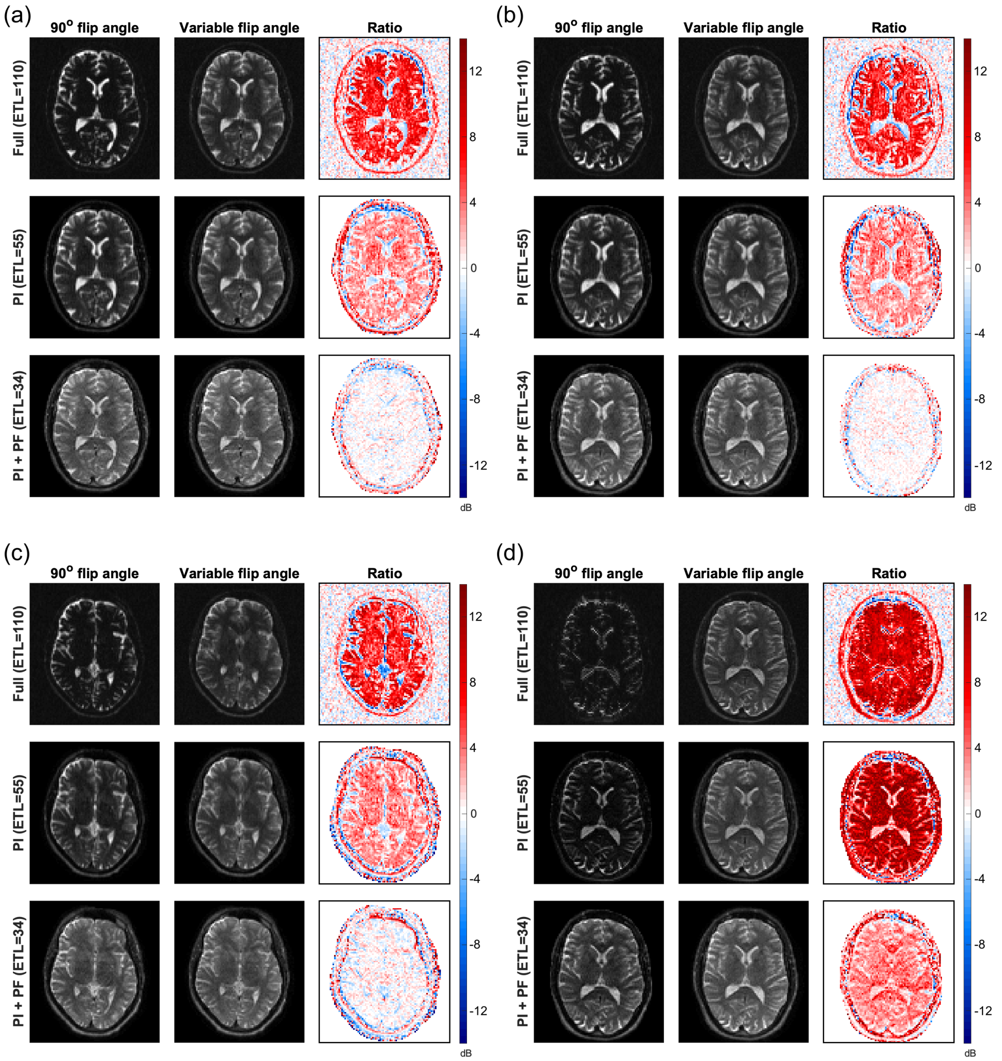

Figure S5: An example slice for the SPLICE b=0 s/mm^2 data scaled with the background noise level for all three scans/subjects. (a): scan 1, linear k-space sampling order (also presented in Figure 4 of the main manucript). (b): scan 2, linear k-space sampling order. (c): scan 3, linear k-space sampling order. (d): scan 2, centric k-space sampling order. All images are presented with a common, arbitrary intensity scale for each row of images. For each subfigure, the fully sampled data is presented in the top row, and the undersampled data in the two bottom rows. Coil sensitivities were estimated only for areas within the subject, so the background is removed for undersampled data.

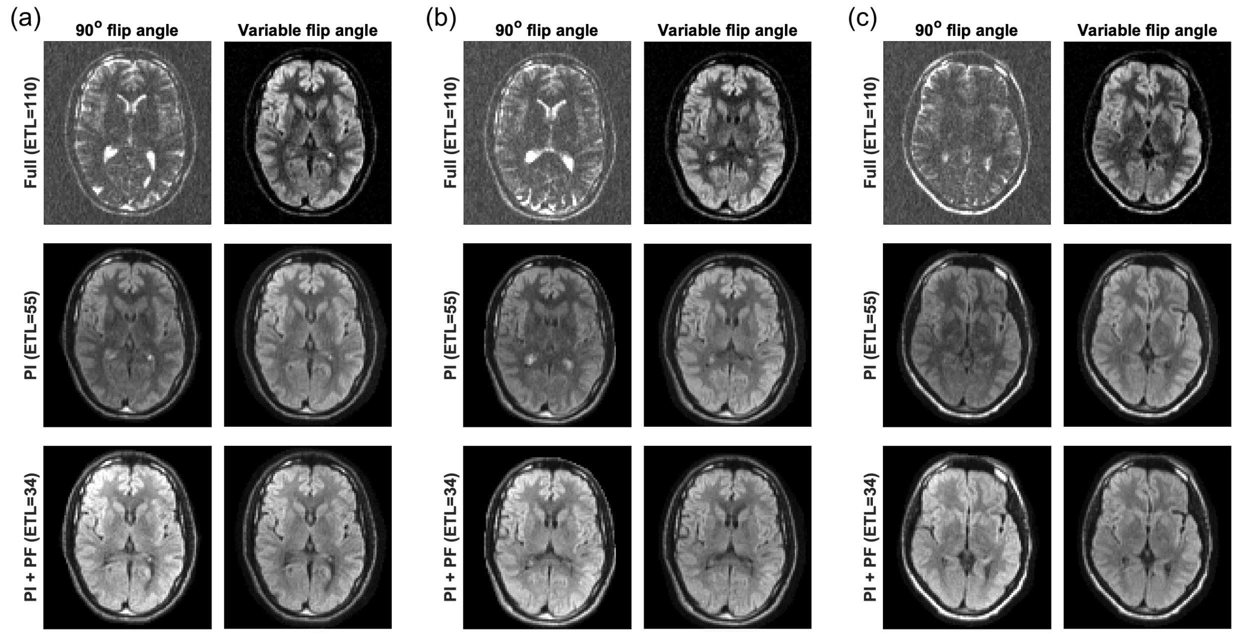

Figure S6: A single slice for the SPLICE b=800 s/mm^2 data for all three scans/subjects (obtained with linear k-space sampling order). (a): scan 1. (b): scan 2. (c): scan 3. Images are presented with a common, arbitrary intensity scale for each row of images. The fully sampled data is presented in the top row, and the undersampled data in the two bottom rows. Coil sensitivities were estimated only for areas within the subject, so the background is removed for undersampled data.

Figure S6 shows an example diffusion-weighted SPLICE image for each of the scan sessions recorded with linear k-space sampling order. The images were reconstructed using the magnitude sum over the four averages and the geometrical mean across directions. The sum-of-squares combination of coil images results in a non-central chi signal distribution, and hence a bias for low SNR data. The resulting ADC maps for the same data is presented in Figure S7. Figure S8 shows similar results and illustrates motion robustness in the base of the brain where motion is unavoidable.

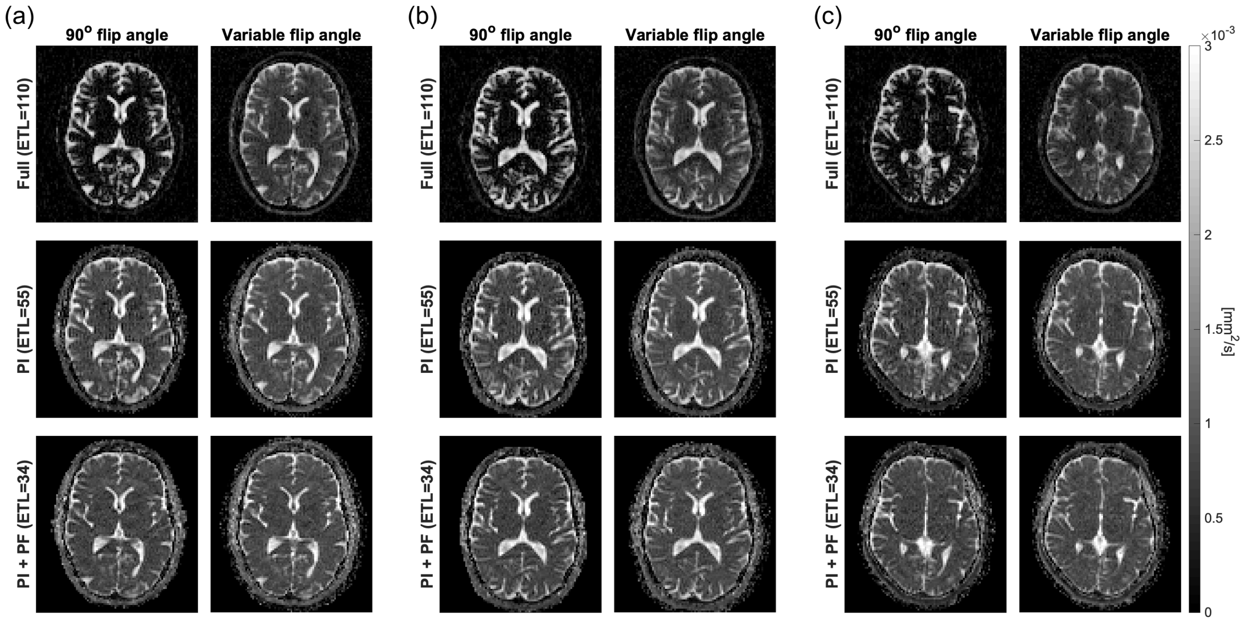

Figure S7: ADC map for a single slice for all three scans/subjects. (a): scan 1. (b): scan 2. (c): scan 3. The fully sampled data is presented in the top row, and the undersampled data in the two bottom rows.

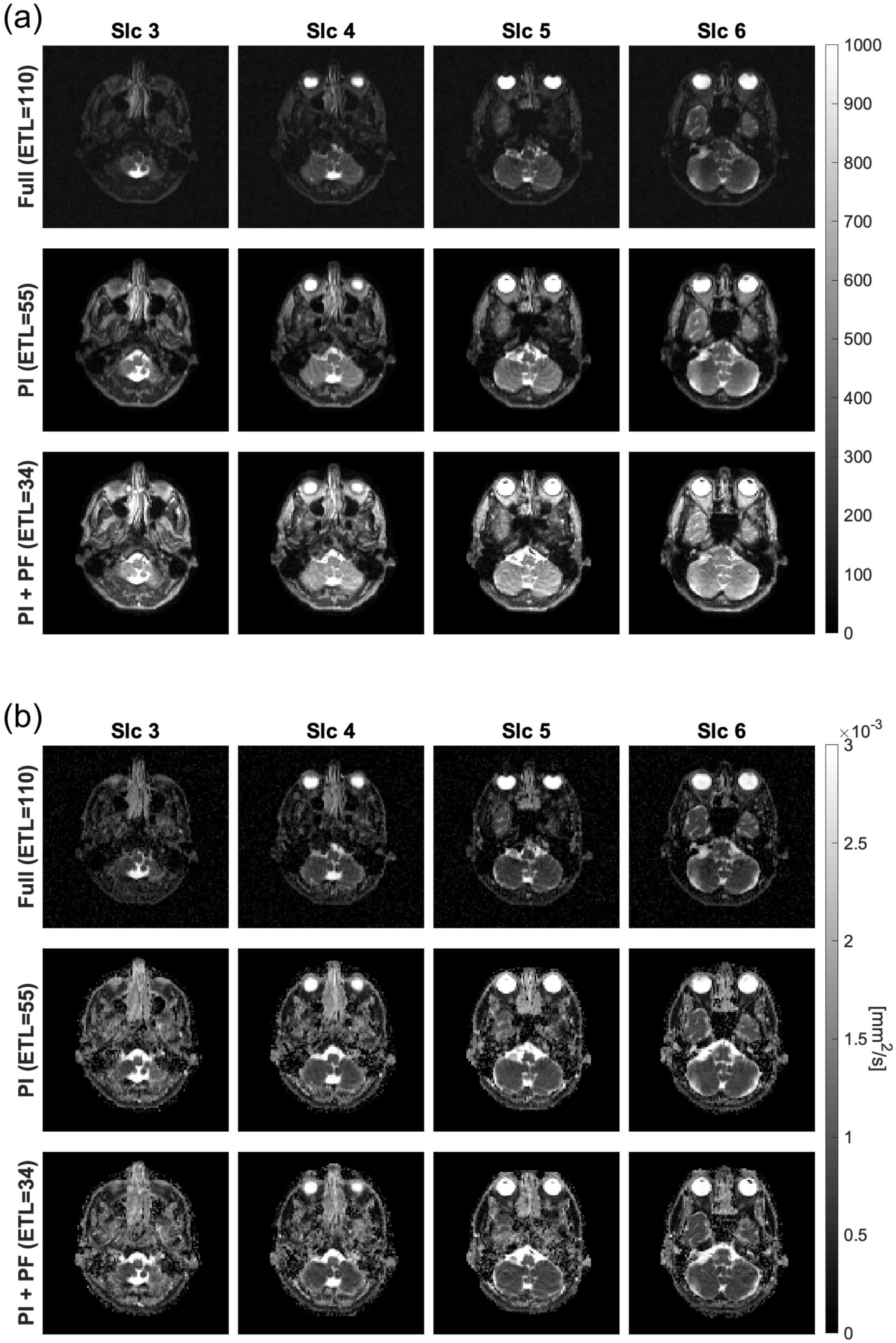

Figure S8: (a) b=0 s/mm^2 images and (b) ADC maps of the lower brain for one example dataset (scan 1, variable flip angle scheme). The fully sampled data is presented in the top row, and the undersampled data in the two bottom rows. Slice 6 (right) is also shown in Figure 6 of the main manuscript.
